# Home Videophones Improve Direct Observation in Tuberculosis Treatment: A Mixed Methods Evaluation

**DOI:** 10.1371/journal.pone.0050155

**Published:** 2012-11-30

**Authors:** Victoria A. Wade, Jonathan Karnon, Jaklin A. Eliott, Janet E. Hiller

**Affiliations:** 1 Discipline of Public Health, The University of Adelaide, Adelaide, Australia; 2 Discipline of General Practice, The University of Adelaide, Adelaide, Australia; 3 Australian Catholic University, Melbourne, Australia; Institut de Pharmacologie et de Biologie Structurale, France

## Abstract

**Background:**

The use of direct observation to monitor tuberculosis treatment is controversial: cost, practical difficulties, and lack of patient acceptability limit effectiveness. Telehealth is a promising alternative delivery method for improving implementation. This study aimed to evaluate the clinical and cost-effectiveness of a telehealth service delivering direct observation, compared to an in-person drive-around service.

**Methodology/Principal Findings:**

The study was conducted within a community nursing service in South Australia. Telehealth patients received daily video calls at home on a desktop videophone provided by the nursing call center. A retrospective cohort study assessed the effectiveness of the telehealth and traditional forms of observation, defined by the proportion of missed observations recorded in case notes. This data was inputted to a model, estimating the incremental cost-effectiveness ratio (ICER) of telehealth. Semi-structured interviews were conducted with current patients, community nursing and Chest Clinic staff, concerning service acceptability, usability and sustainability. The percentage of missed observations for the telehealth service was 12.1 (n = 58), compared to 31.1 for the in-person service (n = 70). Most of the difference of 18.9% (95% CI: 12.2 – 25.4) was due to fewer pre-arranged absences. The economic analysis calculated the ICER to be AUD$1.32 (95% CI: $0.51 – $2.26) per extra day of successful observation. The video service used less staff time, and became dominant if implemented on a larger scale and/or with decreased technology costs. Qualitative analysis found enabling factors of flexible timing, high patient acceptance, staff efficiency, and Chest Clinic support. Substantial technical problems were manageable, and improved liaison between the nursing service and Chest Clinic was an unexpected side-benefit.

**Conclusions/Significance:**

Home video observation is a patient-centered, resource efficient way of delivering direct observation for TB, and is cost-effective when compared with a drive-around service. Future research is recommended to determine applicability and effectiveness in other settings.

## Introduction

The treatment of tuberculosis (TB) requires patients to take multiple antibiotics for a minimum of six months; if medication is taken correctly and completely, success rates for both active and latent TB approach 100% [1]. Failure to complete treatment increases the likelihood of recurrence and of developing drug resistant TB, which requires longer, more expensive, and less effective treatment [2], [3]. The direct observation of patients with TB taking their medication is intended to improve adherence, and is recommended by the World Health Organization (WHO) as part of standardized short-course chemotherapy [4].

Direct observation is resource-intensive for healthcare services and requires time and effort from patients [5], [6]. A Cochrane review containing 11 randomized controlled trials (RCTs) concluded that there was no significant difference in TB cure rates between direct observation and self administration of medication, and recommended that funding spent on direct observation would be better directed to other aspects of TB control [7]. However, only one of these RCTs achieved the WHO treatment target of 85% success rate, indicating poor adherence was an issue in all countries.

The reasons for this low efficacy may lie in the characteristics of each setting. In rural areas of developing countries, patient attendance at a clinic for direct observation takes time and money that the patients often lack, leaving a stark choice between receiving treatment or earning a living [8], [9]. In the USA, direct observation can fail because some environments of high crime and drug abuse are too dangerous for outreach workers [10]. A study in Australia deemed home visiting by nurses to be too expensive, and direct observation by trained family members could only be implemented for 58% of the sample because many patients lived alone [11].

Two systematic reviews of qualitative investigation into the facilitators and barriers to complying with TB treatment found that social, cultural and health system factors such as poverty, stigma, and how treatment and care are organized, reduce the effectiveness of direct observation [12], [13]. Regarding service delivery, it has been asserted that TB services are “rarely designed with users' needs in mind and often did not fit readily into the tempo of people's lives” [12].

The case for universal direct observation of all TB patients is that drug resistance is reduced [14], and adherence cannot be predicted by patient characteristics [15]. Economic analyses have found that direct observation is more expensive than standard medical management [16], [17], but is cost-effective when the relapse rate and cases averted are accounted for [18], [19].

Telehealth, or the delivery of healthcare services at a distance using information and communications technology, has the potential to address several deficiencies in the delivery of direct observation. Using home videophones for real-time direct observation of TB was found to be feasible and acceptable in three pilot studies [20], [21] [22], one of which also found cost savings [20]. One feasibility study in an underdeveloped country showed it was possible to use mobile phones to capture video clips of medication ingestion [23]. In general it has been difficult for telehealth services to move beyond a pilot or trial phase into routine operations [24]–[26].

In 2007, the Royal District Nursing Service of South Australia (RDNS SA), a community nursing service, commenced a telehealth pilot program for medication management [21], installing desktop videophones and broadband data connections in patients' homes. This became a routine service from 2009 [27], operating 24/7 within a larger call center, making daily video calls to patients at mutually agreed times. Previously, direct observation was conducted by daily drive-around home visits, and patients not able to be seen during office hours were usually discharged back to management by TB services.

This paper presents the results of a mixed methods evaluation of the home videophone service, using a convergent parallel approach [28], in which each component of the study was conducted independently, then synthesized at the interpretation phase. The specific objectives were:

- to compare the effectiveness of in-person versus home videophone direct observation as measured by the proportion of missed observations in each group
- to determine the cost-effectiveness of home videophone observations under a range of conditions
- to determine the acceptability, usability and sustainability of the home videophone service by interviewing patients and providers

## Methods

### Ethics

This study was approved by The University of Adelaide Human Research Ethics Committee, the Royal Adelaide Hospital Research Ethics Committee, and the South Australian Department of Health Human Research Ethics Committee. All participants who were interviewed gave written informed consent. Consent was waived by the above ethics committees for retrospective case note access, provided that no individuals were identified, as allowed by the Australian Government National Statement on Ethical Conduct in Human Research [29], on the grounds that the research was low risk and obtaining consent for this component of the study was impractical.

### Quantitative study

#### Design

A retrospective cohort design was used to compare TB patients who had received direct observation by home videophone with patients who had received this service in person, either by a drive-around service or clinic attendance, because the service model had altered from mainly in-person to mainly home videophone delivery from early 2009.

#### Participants

Data were sought from the records of the patients who had received direct observation for TB from RDNS SA, from the beginning of 2003 (when records became readily available), to the 15^th^ November 2010.

#### Data Sources

The uptake of the home videophone service was determined from data provided by the Royal Adelaide Hospital Chest Clinic, showing the total numbers of new TB cases per year commencing treatment, the numbers of patients referred for any form of direct observation, and those specifically referred to RDNS SA.

Within RDNS SA, patients were identified by searching the RDNS SA electronic business system for all patients funded by the Chest Clinic, and all patients with a diagnosis of TB. The paper case notes of these patients were then obtained for data extraction, which was repeated for five percent of cases by the same researcher (VW), after a minimum interval of two weeks, to assess reliability.

#### Data Collected

Age, sex, country of origin, and English-language capability were collected from admission forms. Length of service was determined as the number of days the patients were enrolled for direct observation, excluding any periods for which patients had their medication suspended.

The denominator for assessing effectiveness was the number of occasions on which the patients were supposed to take their medication. Most had a medication regimen of taking their tablets once a day, but a minority was medically required to take their medication on a different schedule, such as three times a week, or twice daily, which is reflected in the number of intended observations.

The numerator for assessing effectiveness was the number of missed observations, recorded if the visit record and the medication chart record together noted an absence, or if a specific mention of non-observation was made in the progress notes. Partial observation, where only some of the tablets taken were observed, was counted as a successful observation. Reasons for missed observations and discharge from the service were also collected and compared by service type.

#### Statistical Analysis

Comparison of the video and in-person groups matched each videophone patient with an optimal match from the in-person service, which could be either whole-person or proportionate matches of several individuals, using the GenMatch automated search algorithm. The balance of the observed covariates was maximized by an iterative process, using a generalized Mahalanobis Distance metric, and was continued until there were 199 iterations without improvement [30]. The resulting matched dataset was bootstrapped using the Stata statistical package [31] to generate 95% confidence intervals around the difference in the outcomes between the comparison groups.

### Economic Analysis

**Figure 1:**
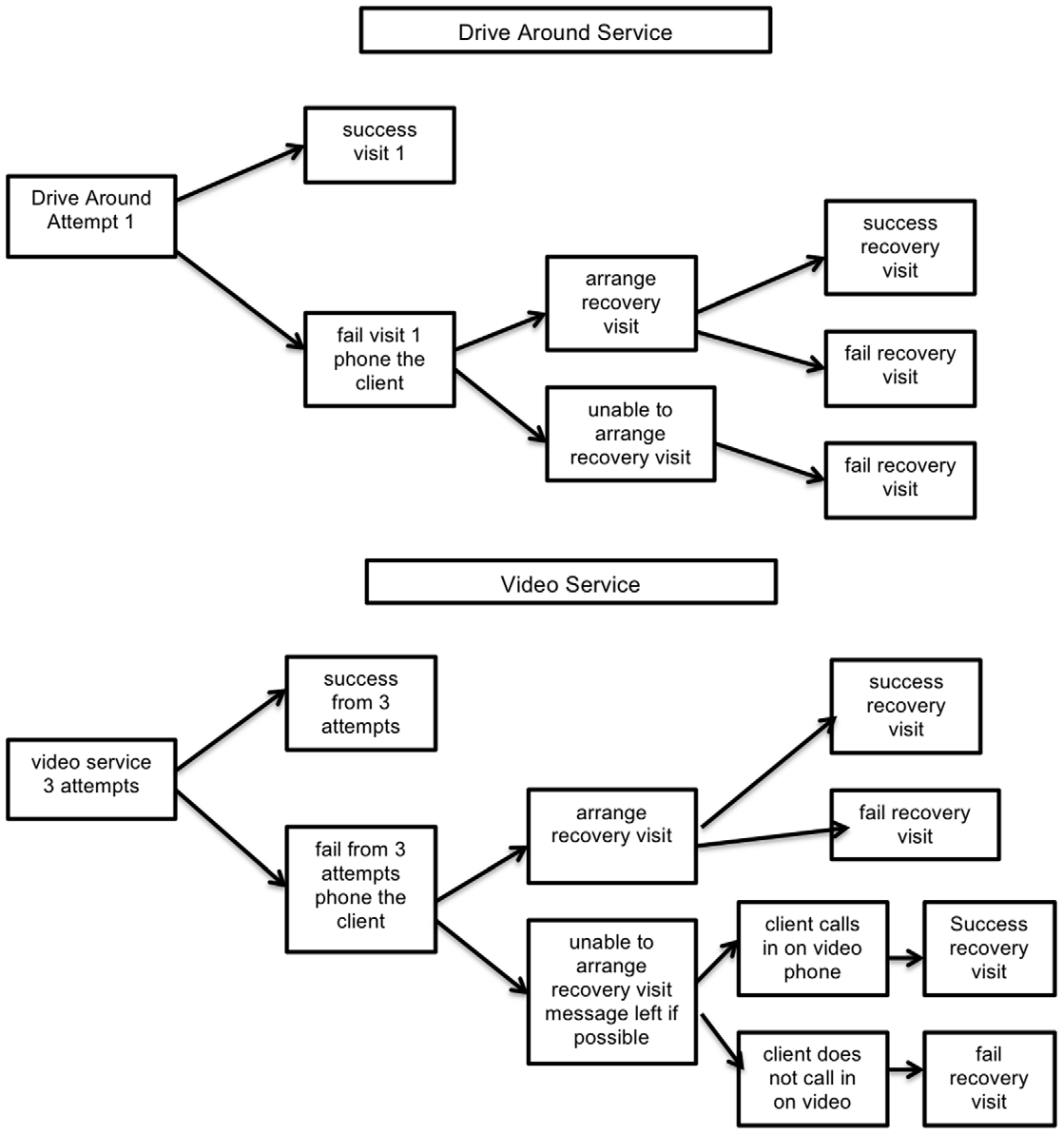
The workflow of delivering direct observation.

To jointly estimate the costs and outcomes associated with a traditional drive around service, compared to a mixed video plus drive-around service, a dynamic cell-based cost-effectiveness model was built in Microsoft Office Excel [32]. The workflow processes which fed into this model were informed by the RDNS SA staff interviews, and are shown in Figure 1. Prior to the videophone service, all clients entered the drive-around service, whereby if the patient is not present at the time of the pre-arranged home visit, an attempt is made to telephone the patient and arrange a second time for direct observation that day. In the videophone service, a proportion of patients are deemed unsuitable for the home videophone option and continue to use the drive-around service. For those using the videophone service, if patients do not answer an initial video call, the service tries twice more to make a video call before telephoning patients and attempting to leave a message. Patients can also initiate a video call, providing an additional avenue for achieving successful observation.

The model was populated to estimate the proportion of visits that follow each of the defined pathways, with costs attached to the events along each pathway. The population of patients entering the service is categorized with respect to an underlying measure of adherence to the traditional drive around form of direct observation: compliant (<20% of observations missed), erratic (20 to 50% of observations missed), and non-compliant (>50% observations missed). The percentages of patients in each adherence group were sourced from the matched in-person patient cohort described in the Quantitative study methods section. For the mixed service option, the proportion of patients continuing to use the drive around service was based on the figure in the current operations of the videophone service.

The following section describes the data and assumptions used in the population of the model. Table 1 presents the full range of parameter values and data sources.

**Table 1:** Economic model input parameter values and sources.

Variable	Values	Source of Data or Estimate
% compliant, erratic and non-compliant patients	36.3, 39.4, 24.3	RDNS SA Case Note Review; matched non-video patients
Number of patients/year on direct observation	47	Royal Adelaide Hospital Chest Clinic
Average trip speed	32 kph	Tranter [43]
Length successful in-person visit	19 mins	RDNS SA business records
Length failed in-person visit	5 mins	Estimate from nurse interviews
Drive time between visits	5 mins	RDNS SA business records
Length successful 1^st^ video visit	8 mins	RDNS SA business records
Length failed 1^st^ video visit	4 mins	Estimate from nurse interviews
Length recovery video visit	7 mins	RDNS SA business records
Length failed recovery video visit	1 min	Estimate from nurse interviews
Supervisor/staff ratio: drive-around	0.071	Estimate from nurse interviews
Supervisor/staff ratio: call centre	0.033	Estimate from nurse interviews
% in-person patients seen on weekends drive-around service	50%	Estimate from case note review
Ratio registered to enrolled nurses drive-around service	0.6	Estimate from nurse interviews
Ratio registered to enrolled nurses video service	0.2	Estimate from nurse interviews
Nurses working hours	38/week	Nurses (South Australia) Award [44]
Nursing salaries	Range: $46K to $85K p.a.*	Nurses (South Australia) Award [44]
Office costs/workstation/yr	$4,800*	Employee cost calculator [45]
Video service costs/unit/month	$200*	Video service provider
Car costs/km	$0.63*	Australian Tax Office [46]

* Currency conversion using purchasing power parity $AUD1 = £UK0.42 [47].

#### Resource Use and costs

Daily resource use was estimated for each model pathway, which were multiplied by the corresponding pathway probabilities and summed to generate an expected cost per patient. Whole of service costs per annum were estimated by multiplying the expected cost per patient by the annual patient prevalence , determined by the number of new cases referred for direct observation per year, and the duration of each case.

The main resource element is nursing time. For the drive around service, nursing time includes the time spent driving between visits. For both service options, alternative times associated with failed and successful observation visits/calls were estimated. Other resources included car running costs (based on estimated average distances between visits), supervisor time, office costs and videophone rental charges. A general assumption is that services are part of larger drive-around and call-centre operations, which allows the allocation of fractional resource units.

Costs were attached to the resources used: salaries for enrolled and registered nurses, additional costs of covering seven days a week, shift work, annual and sick leave, and compulsory on-costs such as superannuation, insurance, and taxes. Proportions of patients seen after-hours, and the proportion of registered versus enrolled nurses were represented. Supervisory staff, office costs, and technology costs were also included in the estimated cost per whole episode of service per patient, and the total cost of the service to the organization per annum. Cost-effectiveness was estimated as the cost per additional day of successful observation of the mixed video with residual drive-around service compared to the drive-around service alone.

#### Calibration, Scenario and Sensitivity Analysis

Probabilistic calibration was used to generate multiple sets of input parameter values for which there was stochastic uncertainty [33]. The model was calibrated to observed data describing the numbers of observation days in separate cohorts of videophone, and drive-around patients, as measured by the case note review. Sampling values from plausible ranges for relevant input parameters, the effectiveness component of the model was run until 100 convergent parameter sets were found that matched the observed model outputs to two decimal places. The mean outputs and 95% confidence intervals across the 100 convergent parameter sets were reported as the reference case results. The convergent parameter sets represent the joint uncertainty in the input parameters, and so the outputs across the convergent sets were analyzed to estimate the probability that the videophone service is cost-effective at different monetary values per additional day of observation, presented in the form of a cost-effectiveness acceptability curve.

A set of deterministic scenario analyses was also undertaken to model the effects of service variables, which were defined as parameters that could be estimated locally in areas that are considering implementing a videophone DOTS service. Two scenario analyses were also performed to model the outcomes in two plausible settings of a larger city and a service run wholly by a TB clinic.

### Qualitative Study

#### Data Sources

Interviews were requested with clinicians delivering TB services at the Chest Clinic, clinical staff and managers associated with the videophone service at RDNS SA, and current RDNS SA patients who had been receiving direct observation via videophone for at least one month. Text was also collected from case notes when this provided additional perspectives on service delivery issues.

#### Recruitment

Patients were recruited by RDNS SA staff asking current patients if they would be interested in receiving information about a research project on the service. Willing patients were posted the information sheet and consent form, and the researcher (VW) made telephone contact for recruitment. Health services staff were recruited by email or direct contact, following permission from service management.

#### Interview Methods

Interviews were semi-structured, containing open-ended questions asking for a description of the participant's experience of the videophone service, the acceptability, usability and quality of the service, preferences for method of service, problems or difficulties, and advice or suggestions. Staff interviews also addressed the development of the service, its effect on clinical service delivery, liaison with other services, and factors affecting sustainability.

#### Data Analysis

Interviews were audio-recorded, transcribed and entered into NVivo software [34]. As the healthcare staff worked in small, easily identified units, they were sent their interview transcripts for review, allowing removal of any part which they did not want used.

A thematic analysis was conducted, taking a realist approach to identifying repeating patterns in the data, connecting these to each other and to concepts in the literature [35]. The themes were related to each other to form a diagrammatic model explaining the reasons for the successful uptake of the videophone service.

## Results

### Quantitative Study

#### Uptake of Direct Observation

The uptake of direct observation in South Australia is shown in Table 2, which combines the RDNS SA data with the Chest Clinic data from 2006.

**Table 2:** Uptake of direct observation by year and method.

Year	2006	2007	2008	2009	2010*
N total TB patients in SA	74	56	58	54	58
N TB patients on direct observation	26	21	26	39	42
% TB patients on direct observation	35.1%	37.5%	44.8%	72.2%	72.4%
N RDNS SA video patients	0	2*	0	37	30
Video % of total TB patients	0%	3.6%	0%	50.0%	51.7%
N RDNS SA in-person patients	11	9	14	5	3
In-person % of total TB patients	14.9%	16.1%	24.1%	9.3%	5.2%

* to Nov 15 **pilot study patients.

The total number of people who commenced treatment for TB was steady over time. Before the videophone service, a minority of patients received direct observation, and of these, less than half were sent to RDNS SA, where they received either a drive-around service or attended a nursing centre. During the period studied, the RDNS SA progressively closed its suburban nursing centres, leaving only the drive-around in-person option by 2010. As only seven patients attended a nursing centre, this group was combined with the drive-around service, to form one in-person group. Following the decision by RDNS SA at the beginning of 2009 to implement telehealth on an ongoing basis, the percentage of patients referred for direct observation has increased, with most being placed into the videophone service.

#### Selection of Records for Case Note Review

The RDNS SA electronic patient database contained 225 records of patients who were either funded by the Chest Clinic or had a recorded diagnosis of TB, between 1^st^ January 2003 and 15^th^ November 2010. Patients were excluded if they were not treated for TB, were receiving intramuscular or intravenous treatment, or if data were missing from their notes, leaving 128 patients who were seen for 132 episodes of direct observation. Four repeat episodes of care were excluded from subsequent analysis because they were not directly comparable to first episodes.

#### Data Extraction Checking

Six sets of notes were recoded, with four exhibiting minor differences; in each case a single day of service was coded differently, and these were checked a third time to determine the final numbers. All extracted raw data was checked a second time for consistency, and where differences were found, a third time. These changes were minor and did not alter the aggregated results.

#### Demographic Characteristics of the Patients

Table 3 shows the patient demographics for videophone and non-videophone patients.

**Table 3:** Patient characteristics.

	Videophone	%	In Person	%	Total
**Gender**					
Male	32	55.2%	46	65.7%	78
Female	26	44.8%	24	34.3%	50
**Age**					
0–19	3	5.2%	8	11.4%	11
20–29	21	36.2%	16	22.9%	37
30–39	19	32.8%	14	20.0%	33
40–49	5	8.6%	13	18.6%	18
50–59	6	10.3%	4	5.7%	10
60+	4	6.9%	15	21.4%	19
**Region of Origin**					
Africa	7	12.1%	12	17.1%	19
Australia	1	1.7%	11	15.7%	12
Europe	2	3.4%	6	8.6%	8
Eastern Asia	9	15.6%	5	7.1%	14
South East Asia	18	31.0%	22	31.4%	40
Southern Asia	21	36.2%	14	20.0%	35
**Proficiency in English**					
Good	40	69.0%	39	55.7%	78
Poor/none	18	31.0%	31	44.3%	49

With the introduction of the videophone service, the percentage of females increased, the age distribution shifted towards the young adult group, a larger percentage spoke English well, and a higher percentage came from the Southern Asia region.

#### Video versus In-Person Service Comparisons

Service comparisons between the videophone and in-person groups are shown in Table 4. The different demographic characteristics of the two groups were compensated by matching, which increased the validity of calculating confidence intervals.

**Table 4:** Service outcome comparisons.

	Videophone (n = 58)	In Person (n = 70)	Mean Difference (95% Confidence interval)
Average length of service (days)	BM 163.3	133.0	
	**AM 163.4**	**116.7**	**46.6 (18.2–76.5)**
Average number of service episodes	BM 158.9	124.1	
	**AM 158.7**	**114.3**	**34.3 (14.1–72.5)**
Average number of non-observations	BM 13.4	40.6	
	**AM 13.5**	**41.0**	**27.5 (16.6–40.0)**
% Service episodes not observed	BM 12.2	31.8	
	**AM 12.1**	**31.1**	**18.9 (12.2–25.4)**
Observation days lost: with permission	BM 2.0	30.0	
	**AM 2.0**	**30.1**	**28.1 (19.3–40.7)**
Observation days lost: non-adherence	BM 5.3	6.4	
	**AM 5.3**	**3.3**	**2.0 (−5.4–4.8)**
Observation days lost: technical problems	BM 2.0	0	
	**AM 2.0**	**0**	**2.0 (1.2–3.0)**
Observation days lost: service provider issue	BM 2.1	0.8	
	**AM 2.1**	**0.7**	**1.4 (1.2–3.0)**
% Patients discharged: treatment complete*	BM 57.7	42.8	
	**AM 47.8**	**32.8**	**14.9 (−0.2–32.6)**
% Patients discharged: moved out of area*	BM 17.8	14.3	
	**AM 15.3**	**23.4**	**8.1 (−2.0–5.2)**
% Patients discharged: to Chest Clinic mgmt*	BM 17.8	35.7	
	**AM 13.7**	**39.4**	**25.7 (9.0–40.1)**

BM = before matching AM = after matching and bootstrapping.

* Comparisons after removing 13 videophone patients who had not yet completed treatment from the sample.

The videophone service was more effective than the in-person service, with a significantly reduced percentage of missed observation episodes. The seven patients who attended a nursing center missed 31.2% of observations compared with 31.9% in the 63 patients receiving a drive-around service, therefore these two groups were combined into a single in-person group.

The main reason for the videophone service reducing missed observations was far fewer days lost to pre-arranged absences, such as weekends, attending medical appointments, being on holidays or at work. The videophone service attempted to see all patients seven days a week, whereas on weekends the nursing centers were closed and the drive-around services were reduced.

Non-adherence, where the patient was absent and could not be contacted, present but refused to take their medication, or claimed they had already taken their medication before the visit, was the same in both types of service. An average of two days was lost from the videophone service due to technical problems, although the variation between patients was large with some having none, and others frequent technical issues. Reasons for technical failure recorded in the case notes included videophone, modem or power failure, poor quality video calls, videophone not responding to rebooting, and connectivity failure, with this latter category subdivided into congestion, internet service provider failure, and landline disconnection. These reasons were not recorded consistently enough to produce reliable subcategories.

The length of service was longer for the videophone group because the service model changed to keep patients on direct observation until the end of their TB treatment. This difference is a conservative estimate because at the time of data extraction, 13 of the videophone patients were still enrolled in the service. This change in approach meant that fewer patients on the videophone service were discharged from direct observation to routine management by the Chest Clinic, and a higher proportion was seen by RDNS SA until their TB treatment was complete.

### Economic Analysis and Model

#### Cost-Effectiveness

Table 5 shows the reference case, in which the model is populated with the RDNS SA data to determine the cost-effectiveness of the two whole-of-service approaches.

**Table 5:** Reference case - Service comparisons and economic analysis.

Type of Service	Video+Residual In Person	In Person	Difference (95% CI)
Patients/year	47		
Days observed/episode of care	141	92	49
**Resource Use/day**			
Staff FTE	0.45	1.05	0.61 (0.58–0.63)
Car hours	0.53	6.98	6.46 (6.34–6.59)
Kilometres driven	13.53	60.45	46.92 (44.74–48.89)
**Costs ($AUD)**			
Whole of service cost/year	$124,753	$121,686	$3,067 (1,184–5276)
Cost/complete patient care episode	$2,654	$2,589	$65.26 (25.20–112.27)
ICER- Cost per additional successful day of observation		$1.32 95% (0.51–2.26)	

The model indicates that operating the video service on this small scale is near to break-even, but is a little more costly than operating a drive-around service, an overall outcome that was confirmed by RDNS SA. Nonetheless the Incremental Cost-Effectiveness Ratio (ICER) is at a level where this additional cost was borne because of the improved effectiveness of the service.

**Figure 2:**
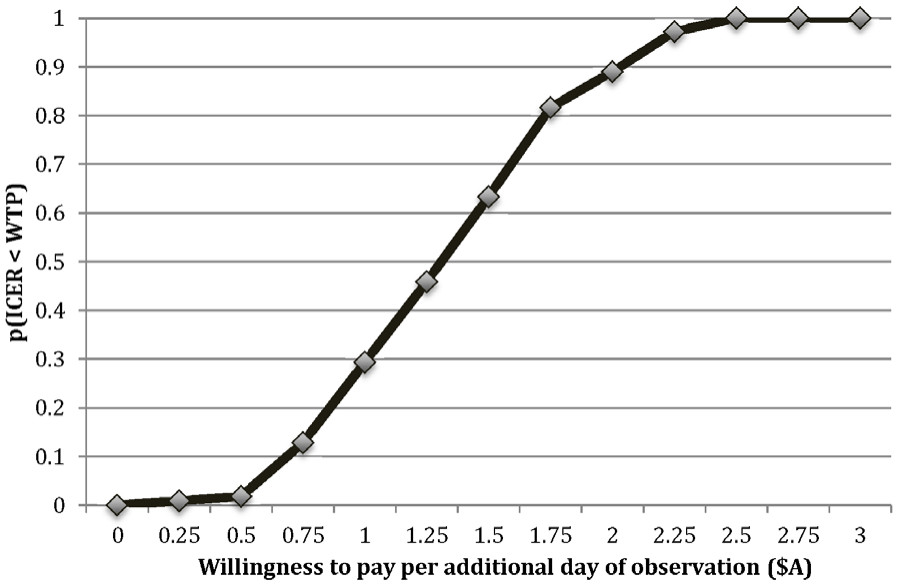
Service comparison cost-effectiveness curve.

The probabilistic calibration generated a distribution of costs and observation days for both comparators, which are presented in Figure 2 in the form of a cost-effectiveness acceptability curve. This shows that if one is willing to pay an additional $1 to gain an additional day of observation, there is a 30% probability that the videophone service is cost-effective, but if one is willing to pay $2, the probability of cost-effectiveness rises to almost 90%.

#### Scenario Analysis

*Scenario 1: Larger city.* Adelaide is a small city with a low prevalence of TB. To model a larger city, client numbers were set at 500 a year, the travel time was increased to 10 minutes between visits at an average speed of 20 kph, (allowing for increased traffic congestion), and the cost of the videophone service was reduced to $150 per month per unit. Under these conditions, four video workstations and 4.3 FTE staff are needed in the call center, together with 222 video units. The cost for the whole of the service favors the video option by $545,716per year, and the ICER indicates dominance, that is, both less costly and more effective.

*Scenario 2: Stand alone service.* To model a TB service with 160 patients a year in a medium-sized city, which is presently conducting its own outreach service, it was assumed that no patients were seen in the evenings, travel time was increased to 15 mins between patients at an average speed of 25 kph (as the patients would be more geographically dispersed, with traffic congestion moderately increased) the videophone costs remain at $200 per month per unit, and the percentage of noncompliant patients is greater at 50%. If it is assumed that fractions of staff and cars could be put to effective use by the organization, then two call center workstations, 1.4 FTE call-center staff and 66 videophone units are needed, with the cost difference favoring video by $198,753 per year and the ICER also indicating dominance.

#### One-Way Deterministic Sensitivity Analyses

Taking the Reference Case as the starting point, the variables that are most relevant to service development were altered one at a time, with the results shown in Table 6.

**Table 6:** One-way deterministic sensitivity analyses.

Variable	Total cost diff/year	Cost diff/patient service episode	Effectiveness difference (days)	ICER
Reference Case	$3,06	$65.2	49	$1.32
Number of patients				
30	$9,348	$311.60	49	$6.30
200	−$30,296	−$151.48	49	dominant
500	−$103,761	−$207.52	49	dominant
% Non-compliant				
10%	$4,305	$91.59	55	$1.63
40%	$5,851	$124.49	43	$2.93
Drive time between patients				
10 mins	−$28,115	−$598.18	49	dominant
20 mins	−$90,478	−$1,925.07	49	dominant
30 mins	−$152,842	−$3,251.96	49	dominant
Cost of technology				
$150/mth/unit	−$11,333	−$241.12	49	dominant
$150/mth/unit	−$25,733	−$547.50	49	dominant
Staff salaries				
↓ by $5,000p.a.	$7,036	$149.71	49	$3.02
↓by$10,000p.a.	$11,170	$237.66	49	$4.80
↓by$15,000p.a.	$15,139	$322.11	49	$6.51
Drive-around weekend service				
All patients	−$23,602	−$502.18	35	dominant
No patients	$27,957	$594.83	63	$9.38
Equal length of service	−$20,905	−$444.79	31	dominant

*Number of Patients.* Reducing the number to a very small service of 30 patients a year makes it more costly but still worth considering, and increasing the numbers while keeping everything else the same leads to modest savings.

*Type of Patients.* Changing the percentage of noncompliant patients makes little difference to the outcome; if the proportion is reduced from the reference case of a 25% of patients to 10%, the video service has small increases in both expense and effectiveness, leaving the ICER unchanged. If noncompliant patients are increased to 40% of the total, there is a small difference in favor of the drive-around service, with more patients who are unsuitable for video being entered into the residual drive-around category.

*Driving Time.* The reference case driving time of 5 minutes between patients was thought to be the minimum possible; as RDNS SA is the major provider of community nursing in Adelaide; each field nurse drives around a geographically compact area. Only an increase was modeled, with any increase in driving time substantially favoring the video service.

*Cost of Technology.* Decreasing the cost of the technology favors the video service by a moderate amount.

*Staff Salaries.* Salary estimates in the reference case may be high compared to nursing costs elsewhere; reducing salaries reduces the total costs of both services substantially, with the balance tipping modestly toward the drive-around service.

*Weekend Service.* The reference case is set at half the drive-around patients receiving a weekend service; reducing this favors the drive-around service because less staff time is used in the hours when it is more expensive. Conversely, if the drive-around service operates for all patients on the weekends it is more costly than the video service.

*Length of Service.* If the drive-around service is continued for the same length of time as the video service, for the whole of the treatment episode, then the video service becomes dominant.

### Qualitative Study

#### Interviews

Thirty interviews were conducted with the four staff at the Chest Clinic who had major responsibility for managing TB patients, 14 RDNS SA staff, and 11 current patients. Eight patients spoke English very well and three had English that was adequate but not fluent, which reduced their ability to express themselves at interview. The technical director of the videophone service provider was also interviewed. The staff interviewees reviewed their transcripts and none requested removal of any material.

#### Thematic Analysis

Analysis of all 30 interviews produced seven themes related to the implementation and effectiveness of the service, shown in Table 7.

**Table 7:** Thematic analysis of videophone service qualities.

Themes	Categories within each Theme
Convenience and flexibility for patients	• Patients could be observed at a time of their choosing, including early morning or evenings, fitting with lifestyle and cultural needs.• Chosen call time delivered reliably; patients did not need to wait.• Patients could initiate a video observation when they were ready.• Patients could change the time of the observation at the last minute.• Patients could move the videophone to another location whenever they chose.
Acceptability for patients	• Rapport with the nurses developed via video contact.• Patients had a positive regard for the technology.• The technology was regarded as very easy to use.• Staff and some patients thought the videophone service was more private than an in-person service. Two patients expressed privacy concerns.
Efficiency for RDNS SA	• Many more patients could be seen in a shift than with a drive-around service.• The service could be initiated rapidly, without technical support.
Technical problems were manageable	• Substantial and ongoing problems with video call quality were very frustrating. The call centre nurses learned to manage most of these themselves.• Occasional whole of system failures were also managed.
Increased patient adherence	• More convenient scheduling was regarded as improving patient adherence.• Absent patients could be readily called back repeatedly.• Patients who had difficulty taking all their tablets at once could be called in stages.• The potential to cheat over the videophone was noticed and protocols instituted to minimize this.
Improved liaison between RDNS SA and the Chest Clinic	• Increased communication about patients occurred.• The Chest Clinic initiated education of call centre nurses• Joint protocol development was undertaken.
Supported by the Chest Clinic	• More patients were referred to RDNS SA for direct observation.• The Chest Clinic encouraged other hospitals to also refer to the videophone service.

Increased convenience and flexibility for patients was a major theme, spoken about by 25 interviewees. Many patients were newly arrived in Australia; nurses noted that the patients had “time management problems, where they've got lots of stress, lots of other difficulties going on” and “are desperate to work, to keep an income going”. The videophone service offered more options:

a lot of them work in jobs where they are out early in the morning, like they start work at seven, so they'll want a call at six in the morning … and the night shift will do that.

The patients could also initiate a video call when they were ready; “after about nine o'clock [pm] we get a lot of them calling in, ‘cause they're nurses or taxi drivers finishing their shift”, and the service was able to meet cultural needs; “for the Muslims, it's really helpful, because we can do it all before sunrise.”

Ten of the 12 patients had a wholly positive attitude to the service, while two expressed mixed feelings. As well as convenience, another factor contributing to acceptability was the good relationship with the nurses. One patient said:

you sort of develop this friendship with the nurses … there are two nurses that I was first introduced to when I was taking my medication, ‘cause when I started mine I was isolated at home, so I was always there for a solid three weeks … they are very caring people.

Most felt that the videophone service improved patients' privacy. For example, a patient said “by videoconferencing I think this is good. Nobody can tell to know, no-one”, but two patients mentioned that the videophone service still felt like an intrusion into their homes. One said he would prefer to visit a clinic because he did not want his children to know that he had TB, however, he had accepted the videophone because the alternative was impractical.

The technology itself was reported as very easy to use, and was regarded positively by patients:

all my family think it's lovely. Some of them come here around the time I take it, and they all stand behind here, and the nurse is there.

In regard to increased efficiency for RDNS SA, as well as the obvious saving of travel time, one nurse reported that it was easier to finish videophone visits, as the patients did not try to prolong calls by offering a cup of tea or social interaction.

However there were frequent, substantial, and ongoing technical difficulties which were a source of great frustration to patients and staff, and interfered with the workflow at the call center. The main problem was temporary freezing and drop out of video calls, which the technical service provider explained was due to variable signal strength because one end of the call was connected via a mobile data service. These problems made calling patients with only basic English particularly difficult. The issues could be ameliorated, but not completely resolved, by finding a location in the house with higher signal strength or installing a higher-gain antenna. Over time the nurses learned to troubleshoot these difficulties; many could be improved by asking patients to reboot their videophones. About once a year the entire videophone network went down and the nurses had to make a decision about each patient as to whether they would call them on the telephone that day, or if a field nurse would be asked to make a drive-around visit.

Both the RDNS SA and the Chest Clinic staff thought that the videophone service improved adherence. Some patients found it hard to take their tablets all at once:

we've had young girls who, it's just a real process; it's a really hard daily struggle for them … sometimes it's the physical struggle of swallowing the tablets. Size and number of tablets; massive amounts.

For these people repeated calls could be made to observe them taking a few at a time. When the service was first initiated, the Chest Clinic raised concerns that patients might find it easier to cheat, and there were a few examples of patients trying to avoid observation recorded in the case notes. One entry read:

Client did not sit down during the call or show me that she was taking the tablets from the bottles. She rattled the pills in the bottles and told me she was taking 8 tablets. It was not apparent that she had 8 tablets between her finger and thumb and appeared to swallow too quickly.

A protocol was developed of the patients needing to stay onscreen, show each tablet, and swallow one after the other. Keeping the patient talking for a short period after swallowing also allowed the nurses to see if there was any difficulty which could have been caused by concealing tablets in the cheek. One nurse noted that there was no additional ability to check adherence in person, as “in this day and age we do not have the right to be sticking things in someone's mouth”. When there were problems with adherence, the Chest Clinic was contacted, and their further discussions with the patients usually resulted in improvement.

As a small number of staff operated the direct observation service, all working in the same call center, there was an unexpected benefit of improved liaison, including joint protocol development, between the RDNS SA and the Chest Clinic. A Chest Clinic staff member said:

There's a lot more communication between RDNS and myself now, as in the home nurses, I had virtually no communication, unless there was an extreme issue.

Another added “I feel a lot more confident with the service and hence I'm not having to do as many home visits”. The Chest Clinic welcomed the increased adherence and better liaison with the nursing service and responded by referring nearly all new TB patients to the videophone service, as well as encouraging other hospitals in Adelaide to do the same. They indicated that this gave them greater control over TB services and made it easier to collect data needed for the national TB control program.

In summary, the advantages and disadvantages of conducting direct observation by home videophone are shown in Table 8.

**Table 8:** Videophone service advantages and disadvantages.

Service Issue	Advantages	Disadvantages
Videophone technology	Easy to operate with minimal training	Frequent problems with call quality
	Home installation can be conducted by a non-technician	Occasional whole of system technical failure
Patient acceptance	Positive attitude to videophones	Nil reported
	Patients and nurses developed rapport via video communication	
Patient adherence	Improved by flexible time and place of delivery	A few instances occurred of patients attempting to fake tablet ingestion
	Improved by repeated call backs	
Patient privacy	Staff and most patients reported improved privacy	Two patients reported a feeling of intrusion into the home
Delivery efficiency	Improved by reducing driving time and visit time	Nil reported
Organizational effects	Improved communication and liaison between services	Nil reported

#### Explanatory Model of Uptake

**Figure 3:**
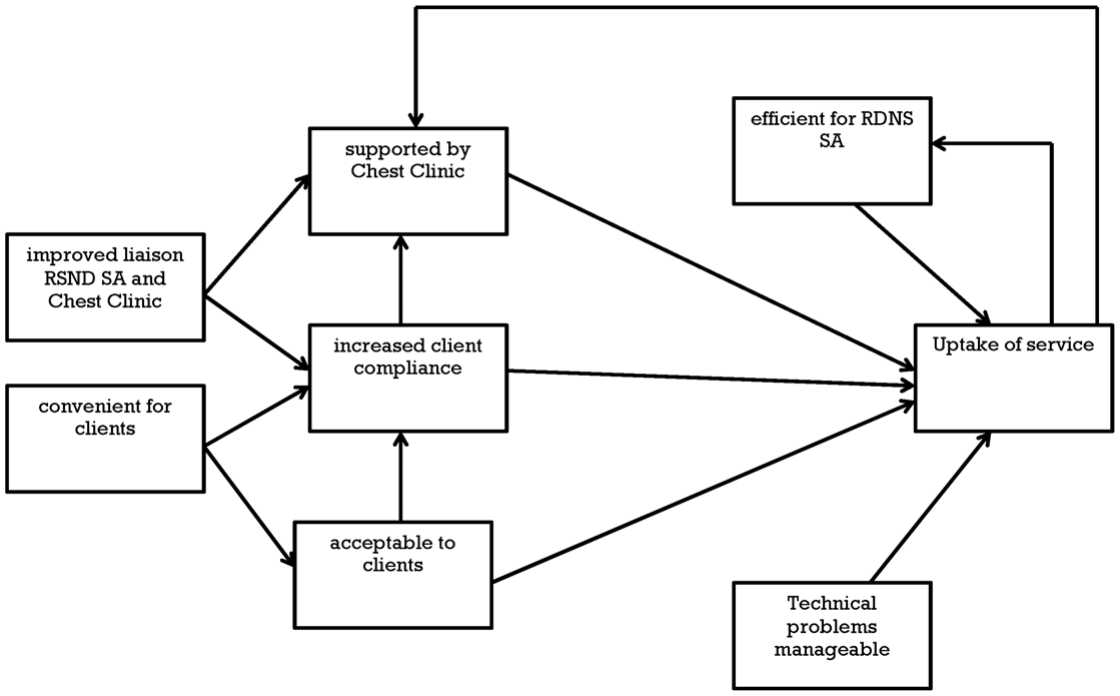
Videophone service uptake model.

Figure 3 shows an explanatory model of these seven themes combining to support ongoing operations, making the videophone service the preferred approach for delivering direct observation. Six the themes were enabling factors, while the major barrier, technical difficulties, could be overcome. The increased uptake produced a positive feedback loop, creating further efficiencies within RDNS SA and more support from the Chest Clinic.

## Discussion

Improving the implementation of TB treatment with direct observation has been framed as a choice between a coercive model which includes universal direct observation, or a patient-centered strategy with communities and healthcare services working together [36], [37]. There is strong advocacy for both approaches, some arguing that direct observation is necessary to achieve high cure rates and prevent drug resistance [38], [39], and others claiming that participatory interventions show greater potential than coercive or inspectorial ones [6], [12], [37], [40], which may drive away those who have most to fear from disclosure [12].

Direct observation has also been difficult to implement; services that are convenient for healthcare staff, such as clinic attendance in office hours, have been impractical, restrictive, and sometimes impossible for patients, whereas outreach services are time-consuming and expensive to operate.

This study suggests that a home videophone approach could offer a means of supplying a high rate of direct observation, although this finding must be tempered by the limitations of the study design, which could not control for all sources of bias. The service was practical to implement in a developed country setting, affordable for the community nursing organization, and was reported by staff and patients to be private, flexible, and convenient. By conducting operations within a 24/7 health call center, it became possible to observe patients after hours, on weekends, or on days when they had other commitments. Staff were readily able to make multiple calls if the patients were absent or unable to take all their tablets at once. Hence many observations which otherwise would have been missed were able to occur.

Nonetheless, the videophone service did not improve the number of observations missed due to patient absence or refusal. The argument could therefore be made that home videophones were simply using a new method to observe people who may have taken their tablets anyway. However the service is intended to be universal, and the qualitative findings indicate how patients, even those who were well-intentioned, well-educated, and living in stable circumstances, struggled with their medications, expressed dislike of taking them, and required considerable support and encouragement to continue treatment. MacIntyre has argued that poor overall compliance rates and inability to predict non-adherence highlights the need for universal direct observation, saying that “interventions to reduce non-compliance are not aimed at patients at the extreme end of the spectrum, but at the majority who are unintentionally non-compliant due to lifestyle factors” [11].

### Limitations

The major weakness of this study was the necessity of using a retrospective cohort comparison between the video and drive-around patient groups. Whilst matching was undertaken for the available data on the demographic characteristics of the patients, other confounding factors may have existed, such as disease severity or socioeconomic status, and the effect of these is unknown.

In regard to the known data, the videophone and non-videophone groups had different demographic characteristics, most likely because the referral criteria to enter the service had broadened to include almost all patients with TB, including university students. This may explain the demographic shift towards young adults with good English from the Southern Asian or East Asian areas. Another possible reason is changing patterns of migration to Australia over time. These differences were taken into account during analysis by matching the samples.

The search for client records within the RDNS SA electronic database may have been incomplete, as the two search methods found different patient lists. However there is no reason to suspect this would have biased the sample. One patient was judged by the nursing service to be unsuitable for the video service, and this exclusion is a potential source of bias in favor of the video service. However as this occurred in only one case, the magnitude of the potential bias would not be sufficient to invalidate the findings.

The study would have been improved by measuring the clinical outcomes of TB, such as cure rate, percentage of relapse, and development of drug resistance, but the study was underpowered to make this comparison; of the 128 cases, only one was an individual who had standard oral treatment for TB and then re-presented with multiple drug resistance; the rate of this outcome is too low to be able to show a significant difference for a sample of this size. Nonetheless, the clinical effectiveness of completion of treatment is well established [1], therefore it is logical that an intervention which improves implementation should also improve outcomes.

Although this study focused on the comparison between the home videophone and a drive-around service, the findings may have broader applicability if a clinic attendance group was added, but the RDNS SA no longer supplied this type of service.

The qualitative study was limited because only videophone patients were interviewed; at the time of this research the single patient receiving a drive-around service was not available for interview. It would have been preferable to arrange interpreters for the three patients whose English was not fluent; their language skills were adequate for basic understanding but some of the nuances of their responses were lost.

The lack of access to confidential internal financial data from RDNS SA is a limitation of the economic analysis, which required staff salaries, office costs, and car costs to be drawn from comparable publicly available data, and some other parameters to be estimated, although the majority of the non-cost aspects were taken from actual service data. The model is a simplification, omitting some of the detailed complexities of daily operations; the aim was to strike a balance between having enough detail to give realistic outcomes, but being broad enough to be usable for services in other settings.

### Acceptability

Patients were largely accepting of the home videophone service, describing a high degree of convenience and flexibility as the main positive attributes. The technology itself was well regarded; whilst technological innovation is sometimes seen as difficult or depersonalizing, this was not the case in this instance. The videophones were an enabler for communication with real people rather than a substitute for personal contact, with both staff and patients describing the development of rapport with each other over time. The technology was easy to use, as the videophones looked and functioned like telephones. There were frustrations from fluctuating poor quality of calls, although this had more effect on the staff than the patients, as it affected their ability to complete their set list of calls on each shift.

A few patients reported that an intrusion on their privacy was a difficulty. These issues would apply to both a video and an in-person service, with the video service usually regarded as offering greater privacy. This is of great value to those patients who experience or are concerned about stigma. The one exception to this is when patients might not want to reveal their status to their family or other people with whom they reside.

The issue of patient autonomy has been prominent in the literature on home telehealth [41], and staff reported that the video service made the patients more independent because they did not have to wait at home for a visit during working hours. However this impression was not confirmed by the patients, who had no experience of the drive-around service; their only comparison was of a freer life before they were diagnosed with TB.

### Economic Viability

The economic analysis indicates that under all conditions the home videophone service saved resources of nursing time, car hours, and kilometres driven. In its current form, the service is cost-effective but not cost saving, however the sensitivity analysis indicates that the videophone service would become dominant with an increase in the size, decrease in technology costs, or increases in driving time between home visits. Under plausible conditions where the videophone service is not dominant, sensitivity analysis modeled the ICER to be at most $10 per additional day successfully observed, which could be considered for implementation by TB treatment services, depending upon their available budgets.

Although the model predicts that less than one FTE staff member can run the entire direct observation service at the current number of 47 patients per year, this was in the context of a larger video medication management service plus general call center, where economies of scale could be realized with several call center staff sharing these duties.

It could be argued that the service would be less costly if the patients supplied their own broadband or mobile data connection, and their own device, such as a home computer, tablet or mobile phone. However in practice, this has a high likelihood of reducing effectiveness and increasing costs, as the quality of the calls would be reduced and a great deal of time taken up providing technical support to patients.

### Sustainability

To the best of our knowledge, this is the first home video direct observation service for TB in the world to have progressed beyond an initial pilot or feasibility test into ongoing, routine operations. The qualitative analysis of the video service indicated that multiple factors contributed to this outcome; particularly demand from the main referrer, efficient use of resources, and acceptability to patients and staff.

It was also important that technical difficulties could be overcome. In the pilot phase the patients had a fixed broadband line installed at home [21]. This was not available in all areas, there was a delay of one to three weeks for activation, and if the patient moved it was difficult to move the connection in a timely manner. The technical development of video calls over the 3G data network drove uptake by enabling the call center nurses to do immediate installations, and although this system was not perfect the technical provider worked with the nurses until they could do most of their own troubleshooting. Hence the call center was able to become reasonably self-sufficient in dealing with the most common technical problems.

Finally, the service proved to be resilient by surviving a number of administrative, structural and governance changes at RDNS SA, although it has remained a small scale operation as numbers of people with TB in South Australia are low. While it provides an example of a sustainable telehealth service, it is a very specific application for a tightly defined purpose, so the results have limited generalizability to the broader uptake and sustainability of telehealth services.

### Risk Analysis

As the health system comes to rely more on technological solutions, the risk of consequences from technical failure grows. The videophone service experienced one shutdown that lasted a week, due to failure of the local telecommunications backbone, and there were several other episodes when the whole network was unavailable for a day or less. With larger patient numbers it would be useful to have a protocol to identify those patients for whom a physical visit should be considered when this occurs.

At the home end of the service, there is a risk of poor quality video calls due to increasing traffic on the 3G data network. However, at least in Australia, the development and installation of new mobile telecommunications infrastructure is keeping pace with this growth in use. In theory, the videophones might be lost, damaged, sold or stolen, but this did not actually occur; these risks would be greater if the patients were loaned mobile phones or tablet computers with video-calling capability.

### Future Research and Development

Continuing technical innovations are expected into the future. Most new mobile (cell) phones and tablet computers can make video calls, although the quality is poor under mobile conditions; the home videophones required dedicated antennas and other technical developments to bring the signal strength and call quality into the acceptable range.

If identified technical issues are solved, video observation could be expanded to the developing world, where mobile technology is the major way that telecommunications infrastructure is being installed, mobile phone penetration is rising rapidly, and low income earners are paying a substantial percentage of their income on telecommunications [42]. Under these conditions, offering to pay for the patients' mobile phone costs might be a strong inducement to participate in direct observation.

A larger study, preferably designed as a randomized controlled trial, and collecting cure and relapse outcomes, should be the next step in assessing this new approach to direct observation, and would contribute substantially to determining whether or not most services could or should move to this model over time.

### Implications for Practice

This method of direct observation can be readily scaled up, especially in larger cities and regions where the telecommunications infrastructure is adequate. Scarce resources can be conserved by seeing larger numbers of TB patients with the same amount of staff. A home video service operated by a specialist TB clinic during office hours would produce some increase in flexibility, but the full benefit would be gained by placing the service within a 24/7 call center.

Additionally, rural patients could be seen, or one call center could serve several cities. The videophones can be posted to patients, who could do their own installation with the aid of an instruction sheet plus a telephone call from the service. There does need to be capability to do hands-on installations for patients with limited English and/or no technical skills at all, but a distant call center could work with local TB treatment services to enable this.

Whether or not this approach is affordable in other jurisdictions will depend upon local health budgets and circumstances. Although the video service was cost-effective compared to a drive-around service, it may still cost more than many TB services can afford; even in a developed country like Australia, a universal drive-around service has not been implemented in all areas because of expense [11]. Finally, the method can be utilized for other clinical conditions where direct observation improves adherence.
